# Chronic exposure to arsenic, LINE-1 hypomethylation, and blood pressure: a cross-sectional study in Bangladesh

**DOI:** 10.1186/s12940-017-0231-7

**Published:** 2017-03-07

**Authors:** Khaled Hossain, Takehiro Suzuki, M. M. Hasibuzzaman, Md. Shofikul Islam, Atiqur Rahman, Sudip Kumar Paul, Tanzina Tanu, Shakhawoat Hossain, Zahangir Alam Saud, Mashiur Rahman, Farjana Nikkon, Hideki Miyataka, Seiichiro Himeno, Keiko Nohara

**Affiliations:** 1 Department of Biochemistry and Molecular Biology, University of Rajshahi, Rajshahi-6205, Bangladesh; 2 Center for Health and Environmental Risk Research, National Institute for Environmental Studies, Tsukuba 305-8506, Japan; 3 Department of Applied Nutrition and Food Technology, Islamic University, Kushtia-7003, Bangladesh; 4 Exim Bank Agricultural University, Chapainawabganj, Bangladesh; 5 Laboratory of Molecular Nutrition and Toxicology, Faculty of Pharmaceutical Sciences, Tokushima Bunri University, Tokushima 770-8514, Japan

**Keywords:** Arsenic, LINE-1, Hypomethylation, Blood pressure, Hypertension, Bangladesh

## Abstract

**Background:**

Chronic exposure to arsenic is associated with cancer and hypertension. Growing evidence suggests that altered methylation in long interspersed nuclear element-1 (LINE-1) is involved in many types of disorders, including cardiovascular disease. Here we evaluated the association between arsenic exposure and LINE-1 methylation levels, especially in relation to blood pressure (BP).

**Methods:**

A total of 236 subjects (175 from arsenic-endemic areas and 61 from a non-endemic area) in rural Bangladesh were recruited. The subjects’ arsenic exposure levels (i.e., drinking water, hair and nail arsenic concentrations) were measured by inductively coupled plasma mass spectroscopy. The subjects’ LINE-1 methylation levels were determined by pyrosequencing.

**Results:**

The average LINE-1 methylation levels of the subjects living in the arsenic-endemic areas were significantly (*p* < 0.01) lower than those of the subjects living in the non-endemic area. In a sex-stratified analysis, the arsenic exposure levels in female but not male subjects showed a significant inverse association with LINE-1 methylation levels before (water arsenic: *p* < 0.01, hair arsenic: *p <* 0.05, nail arsenic: *p* < 0.001) and after (water arsenic: *p* < 0.01, hair arsenic: *p <* 0.05, nail arsenic: *p* < 0.001) adjustment for age, body mass index and smoking. Analyses examining interactions among arsenic levels, BP and LINE-1 methylation showed that arsenic-related elevated levels of BP were associated with LINE-1 hypomethylation.

**Conclusions:**

Our findings demonstrated that chronic exposure to arsenic was inversely associated with LINE-1 methylation levels in blood leukocyte DNA and this was more pronounced in females than males; in addition, the decreased levels of LINE-1 methylation might be involved in the arsenic-induced elevation of BP.

**Electronic supplementary material:**

The online version of this article (doi:10.1186/s12940-017-0231-7) contains supplementary material, which is available to authorized users.

## Background

Arsenic is a potent environmental pollutant and a class I human carcinogen. Chronic exposure to arsenic is a major threat to the public health in many countries, including Bangladesh. Chronic exposure to arsenic has been associated with several neoplastic and non-neoplastic diseases [1–3]. It is estimated that approx. 80–100 million people in Bangladesh are at risk of arsenic toxicity because they are consuming arsenic through drinking water at concentrations greater than the permissive limit (<10 μg/L) set by the World Health Organization (WHO). The arsenic crisis in Bangladesh has been described as the largest mass poisoning of a population in history [4].

Health outcomes of chronic exposure to arsenic are influenced by several genetic and non-genetic factors. The recent development of fine and robust epigenetic techniques and the growing evidence that supports the involvement of epigenetic modifications in cancer and other diseases have created great interest in potential epigenetic biomarkers of arsenic-related diseases. Epigenetic changes, especially changes in DNA methylation have been reported to be implicated in the pathogenesis of many diseases [5–9].

DNA methylation is a chemical modification of the genome that involves the covalent addition of a methyl group (mainly to cytosine residues located in CpG dinucleotides), which converts cytosine to 5-methyl cytosine. Most of the methylation of CpG islands in mammalian genomes is found in transposon elements that include DNA transposons, retrotransposons and endogenous retroviruses. Sequences of these transposable elements may interfere with the regulation of gene expression and genome structure by means of insertions, deletions, and inversions and the translocations of genomic sequences. However, it is believed that transposon activities are decreased by high levels of CpG methylation in transposable elements, which effectively silences these repetitive regions [10, 11].

Long interspersed nuclear element-1 (LINE-1) is a member of LINE family of non-long-terminal retrotransposons, and it is the most abundant retrotransposon found in the human genome, accounting for 20% of the human genome. Growing evidence suggests that hypomethylation in repetitive sequences of DNA such as LINE-1and ALU is associated with various pathophysiological conditions including cardiovascular disease (CVD) [5, 12–17]. Elevated blood pressure (BP) or hypertension is a potent risk factor for CVD, which is the main cause of mortality worldwide. We and other groups have shown that chronic exposure to arsenic is associated with hypertension and circulating biomarkers of CVD [18, 19].

Several population-based studies have identified a link between arsenic exposure and global methylation status; however, the results of these studies are not consistent [14, 20–32]. Most of the previous studies did not show the pathophysiological relevance of the arsenic exposure-related altered DNA methylation status, and thus the pathophysiological consequences of arsenic-related changes in the DNA methylation status have remained poorly understood. The main objective of this study was to explore the association between arsenic exposure and LINE-1 methylation levels recruiting human subjects from arsenic-endemic and non-endemic areas in rural Bangladesh. We also wished to examine whether the altered levels of LINE-1 methylation were linked to arsenic-related BP.

## Methods

### Study areas and subjects

Ethical permission was obtained from The University of Rajshahi, Institute of Biological Sciences, Bangladesh (#32/320-IAMEBBC/IBSc) and National Institute for Environmental Studies in Japan (#2013-2R). The human subjects who participated in this study gave their written consent. The arsenic-endemic and non-endemic areas and study subjects were selected as described [18, 33–38]. Arsenic-endemic study areas were selected from the north-west region of Bangladesh that included Marua in Jessore, Dutpatila, Jajri, Vultie and Kestopur in Chuadanga and Bheramara in Kushtia districts. Chowkoli, a village in Naogaon district with no history of arsenic contamination was selected as the non-endemic area. Adults (18–60 years old) who had lived for at least the last 5 years in the arsenic-endemic or non-endemic areas were recruited.

We attempted to match as much as possible the age, sex and socioeconomic parameters of the arsenic-endemic and non-endemic subjects. The ratio of endemic and non-endemic subjects was approx. 3:1, and the male-to-female ratios in both the endemic and non-endemic areas were approx. 1:1. The subjects in both the arsenic-endemic and non-endemic areas were villagers, and the socioeconomic parameters such as occupation, monthly income and education levels were very closely matched among the areas. The subjects were asked to convene at a convenient location in their respective areas (irrespective of the visible presence of skin symptoms) for enrollment and initial screening purposes. Many people in the arsenic-endemic areas were found to have skin lesions such as melanosis, hyperkeratosis and hard patches on the palms of the hand and soles of the feet. The individuals who did exhibit symptoms were first identified by a physician, and were then confirmed by a dermatologist. The physician carefully examined various parts of the body to confirm the presence of skin lesions caused by exposure to arsenic.

Pregnant and lactating women, individuals who were hepatitis B-positive, and individuals with a history of drug addiction, chronic alcoholism, prescription for hepatotoxic or antihypertensive medication, malaria, kala azar (leishmaniasis), or hepatic, renal or cardiac diseases were excluded from the study. An interview of each subject was carried out by the trained members of our research team who visited each household and used a standard questionnaire. Information obtained from the interview included the sources of water for drinking and daily household uses, water consumption history, socioeconomic status, occupation, food habit, cigarette smoking habit, alcohol intake, personal and family medical histories, history of diseases, physiological complications, previous physician’s reports, and body mass index (BMI).

### Water collection and arsenic analysis

Water samples were collected from the tube wells which the subjects used as a primary source of drinking water, as described [33]. The total arsenic concentration in water samples was determined by inductively coupled plasma mass spectroscopy (ICP-MS) after the addition of a solution of yttrium (10 ppb in 1.0% nitric acid) as an internal standard for the ICP-MS analysis. The accuracy of the ICP-MS determination of the water arsenic concentrations was confirmed by using ‘River water’(NMIJ CRM 7202-a No.347; National Institute of Advanced Industrial Science and Technology, Japan) as a certified reference material (CRM). The average value (mean ± SD) of arsenic in the ‘River water’ determined in triplicate by an ICP-MS analysis was 1.06 ± 0.04 μg/L (reference value, 1.18 μg/L).

### Hair and nail collections and arsenic analysis

Hair and toe nails of the subjects were collected and washed as described [33]. The washed samples were allowed to dry at 60 °C overnight and then digested with concentrated nitric acid using a hot plate at 70 °C for 15 min and 115 °C for 15 min. After cooling, the samples were diluted with 1.0% nitric acid containing yttrium (10 ppb). The concentrations of arsenic and yttrium in these samples were determined by ICP-MS (HP-4500, Agilent Technologies, Kanagawa, Japan). All samples were determined in triplicate and the average values were used. The accuracy of the arsenic measurement was verified by using the CRM“human hair” (GBW09101, Shanghai Institute of Nuclear Research Academia Sinica, China). The average value of arsenic in “human hair” determined in triplicate followed by an ICP-MS analysis was 0.61 ± 0.12 μg/g (reference value, 0.59 μg/g).

### BP measurement

The WHO standard protocol for measuring BP was used. After the subject had rested for ≥20 min, both systolic and diastolic blood pressures (SBP and DBP) were measured three times with a mercury sphygmomanometer with the subject sitting. SBP and DBP were defined at the first-and fifth-phase Korotkoff sounds, respectively. The average of three measurements was used for the analysis. Hypertension was defined as an SBP value of ≥140 mmHg and a DBP value of ≥90 mmHg on three repeated measurements.

### Blood collection and extraction of DNA

Peripheral blood was collected from each subject as described [33]. Fasting blood samples (5–7 mL) were collected in EDTA-containing blood collection tubes from each individual by venipuncture as described [18, 37]. From the EDTA-treated blood, 100 μl was taken and DNA was extracted with the use of a DNeasy Blood and Tissue kit (Qiagen, Valencia, CA, USA) according to the manufacturer’s recommendations. Extracted DNA was kept at −20 °C. Because whole blood DNA was predominantly derived from leukocytes, we refer to the whole blood DNA as leukocyte DNA here in.

### Measurement of LINE-1 DNA methylation

Genomic DNA was digested with EcoRI. The bisulfite conversion of DNA was carried out using the EZ-96 DNA Methylation-Gold™ kit (Zymo Research, Irvine, CA) according to the manufacturer’s recommendations. We conducted a polymerase chain reaction (PCR) amplification of bisulfite-modified DNA using a set of forward primer 5′-TTTTGAGTTAGGTGTGGGATATA-3′ and reverse biotinylatedprimers5′-Biotin-AAAATCAAAAAATTCCCTTTC-3′ as reported [24]. The conditions were as follows: 95 °C for 5 min, and then 50 cycles of 95 °C for 30s, 50 °C for 30s, and 72 °C for 45 s, and finally 72 °C for 5 min.

To measure the methylation level of each of the first three CpG sites next to the pyrosequencing primer, we performed sequencing of the PCR product by pyrosequencing using the PyroMark Q96 ID System (Qiagen) according to the manufacturer’s recommendations. The pyrosequencing primer for LINE-1 was 5′-GGGTGGGAGTGAT-3′. We extracted the methylation level at each CpG site by using PyroMark Q96 software, ver.2.5.8 (Qiagen). The average methylation level of the first three LINE-1 CpG sites was used as the LINE-1 methylation level.

### Statistical analyses

The statistical analyses were conducted with the Statistical Package for the Social Sciences (SPSS ver. 21.0, SPSS, Chicago, IL). A *p*-value <0.05 was considered significant. The normality of the distribution of variables was verified by a Q–Q plot. Because of the skewed distributions of the arsenic exposure metrics, we used log-transformed (natural log) values for the statistical analysis. The differences in descriptive characteristics, arsenic exposure levels and other characteristics between the residents of the arsenic-endemic and non-endemic areas were analyzed by an independent sample *t*-test for continuous variables and the Chi-square test for categorical variables. A nonparametric Kruskal-Wallis test was used to analyze the differences in inter-quartile range (IQR) between the subjects in the arsenic-endemic and non-endemic areas.

We performed a multiple linear regression analysis to examine the effects of age, sex, BMI and smoking on the association between arsenic exposure metrics and LINE-1 methylation levels. To determine the association and the effects of the variables, we obtained the unadjusted R^2^ and adjusted R^2^ values. ΔR^2^ values were calculated by subtracting the unadjusted R^2^ from the adjusted R^2^. Finally, we performed multivariate regression analyses to examine the interactions between arsenic exposure, BP and LINE-1 methylation, and to assess the effects of age, sex, BMI and smoking on the associations of arsenic exposure and LINE-1 methylation levels with BP.

## Results

Table 1 summarizes the descriptive characteristics of the subjects in the arsenic-endemic (*n* = 175) and non-endemic areas (*n* = 61). Of the 236 subjects, 125 were males and 111 were females. Since we attempted to match the subjects’ age, sex and socioeconomic parameters (occupation, monthly income and education) between the residents of the arsenic-endemic and non-endemic areas, no significant differences were observed in those parameters between the two groups. Most of the male subjects were farmers, and most of the female subjects were housewives. We did not identify any female smokers, as generally Bangladeshi women do not smoke. None of the subjects drank alcohol because of the social and religious restrictions on alcohol in Bangladesh.

**Table 1:** Demographic characteristics of the study populations in arsenic-endemic and non-endemic areas

Parameter	All	Non-endemic	Arsenic-endemic	*p*-value
Subjects (n)	236	61	175	
Sex (n)				
Male	125	33	92	
Female	111	28	83	
Age (yrs.)^a^	35.69 ± 10.13	33.92 ± 9.18	36.30 ± 10.40	0.114^*^
IQR	28.00–43.74	27.00–40.00	28.00–45.00	0.079^‡^
BMI (kg/m^2^)^a^	20.94 ± 3.57	20.76 ± 2.77	21.00 ± 3.82	0.655^*^
IQR	18.54–22.57	18.71–22.51	18.37–23.00	0.922^‡^
Occupation [n, (%)]				
Male				0.795^†^
Farmers	97 (77.60)	24 (72.70)	73 (79.30)	
Business	5 (4.00)	1 (3.00)	4 (4.30)	
Students	5 (4.00)	2 (6.10)	3 (3.30)	
Worker	10 (8.00)	4 (12.30)	6 (6.50)	
^+^Others	8 (6.40)	2 (6.10)	6 (6.50)	
Female				0.194^†^
Housewives	105 (94.60)	25 (89.3)	80 (96.40)	
Students	4 (3.60)	2 (7.10)	2 (2.40)	
^‡^Others	2 (1.80)	1 (3.60)	1 (1.20)	
Education [n, (%)]				
No formal education	142 (60.20)	31 (50.82)	111 (63.43)	0.138^†^
Primary	66 (28.00)	18 (29.51)	48 (27.43)	
Secondary	19 (8.10)	8 (13.11)	11 (6.30)	
Higher	9 (3.80)	4 (6.60)	5 (2.90)	
Income/month (US$)^a^	23.45 ± 10.41	22.67 ± 8.89	23.72 ± 10.89	0.457^*^
Smoking in male [n, (%)]				
Yes	43 (34.40)	13 (39.40)	30 (32.60)	0.309^†^
No	82 (65.60)	20 (60.60)	62 (67.40)	
Alcohol intake	–	–	–	–

BMI was calculated as body weight (kg) divided by height squared (m^2^)

*Abbreviation*: *As* arsenic, *BMI* body mass index, *IQR* inter-quartile range

^a^mean ± SD

*p-,^‡^ *p*-and^†^ *p*-values were obtained by an independent sample *t*-test, Kruskal-Wallis test and Chi-square test, respectively. ^+^Others included village doctor, security guard, banker and worker. ^‡^Others included farmer

Table 2 shows the arsenic exposure levels and other characteristic of the subjects. The average concentrations of arsenic in the drinking water, hair and nails of the subjects in the arsenic-endemic areas were approx.70, 8 and 6 times higher, respectively, than those of the subjects in the non-endemic area. The comparison of the arsenic exposure levels between the males and females living in the arsenic-endemic areas showed that the average concentrations of arsenic in the drinking water, hair and nails were slightly higher in the females than the males, although the differences were not significant. Approximately 75% of the subjects in the arsenic-endemic areas showed skin symptoms, whereas none of the subjects in the non-endemic areas did.

**Table 2:** Arsenic exposure levels and other characteristics of the subjects in the arsenic-endemic and non-endemic areas

Parameter	All	Non-endemic	Arsenic-endemic	*p*-value
Subjects (n)	236	61	175	
Drinking water As (μg/L)^b^				
Total subjects	17.76 (15.16)	0.76 (5.49)	53.39 (7.90)	<0.001^*^
Males	13.28 (19.90)	0.60 (4.96)	40.22 (9.02)	<0.001^*^
Females	24.66 (13.97)	0.98 (6.10)	73.09 (6.52)	<0.001^*^
*p*-value (between male & female groups)	0.081^*^	0.269^*^	0.056^*^	
Hair As (μg/g)^b^				
Total subjects	1.28 (4.43)	0.25 (2.38)	2.08 (3.54)	<0.001^*^
Males	1.08 (4.80)	0.19 (2.15)	1.99 (3.70)	<0.001^*^
Females	1.39 (3.99)	0.36 (2.40)	2.20 (3.38)	<0.001^*^
*p*-value (between male & female groups)	0.191^*^	<0.01^*^	0.604^*^	
Nail As (μg/g)^b^				
Total subjects	3.40 (3.53)	0.96 (2.43)	5.29 (2.96)	<0.001^*^
Males	3.19 (3.41)	1.05 (2.32)	4.73 (3.00)	<0.001^*^
Females	3.67 (3.78)	0.85 (2.58)	5.98 (2.98)	<0.001^*^
*p*-value (between male & female groups)	0.403^*^	0.367^*^	0.155^*^	
Skin symptoms [n, (%)]				
(+) symptom	132 (55.90)	0 (0.0)	132 (75.40)	<0.001^†^
(−) symptom	104 (44.10)	61 (100.0)	43 (24.60)	
DBP (mm Hg)^a^				
Total subjects	77.11 ± 11.84	71.97 ± 9.89	78.90 ± 11.96	<0.001^*^
Males	76.73 ± 10.51	73.33 ± 8.26	77.95 ± 10.99	<0.05^*^
Females	77.54 ± 13.21	70.36 ± 11.46	79.96 ± 12.93	<0.01^*^
*p*-value (between male & female groups)	0.608^*^	0.245^*^	0.269^*^	
SBP (mm Hg)^a^				
Total subjects	118.72 ± 18.01	111.18 ± 14.22	121.35 ± 18.48	<0.001^*^
Males	116.56 ± 14.83	112.88 ± 10.91	117.88 ± 15.83	0.097^*^
Females	121.16 ± 20.83	109.18 ± 17.30	125.20 ± 20.45	<0.001^*^
*p*-value (between male & female groups)	0.055^*^	0.334^*^	<0.01^*^	
Hypertension [n, (%)]				
Total subjects				
Yes	32 (13.60)	3 (4.90)	29 (16.60)	<0.05^†^
No	204 (86.40)	58 (95.10)	146 (83.40)	
Males				
Yes	10 (8.00)	0 (0.0)	10 (10.90)	<0.05^†^
No	115 (92.00)	33 (100.0)	82 (89.10)	
Females				
Yes	22 (19.80)	3 (10.70)	19 (22.90)	0.162^†^
No	89 (80.20)	25 (89.30)	64 (77.10)	
*p*-value (between male & female groups)	<0.01^†^	0.054^†^	<0.05^†^	
LINE-1 Methylation level (%)^a^				
Total subjects	64.94 ± 2.12	65.67 ± 1.99	64.69 ± 2.12	<0.01^*^
Males	65.21 ± 1.91	65.46 ± 2.24	65.12 ± 1.78	0.430^*^
Females	64.65 ± 2.33	65.92 ± 1.66	64.23 ± 2.38	<0.01^*^
*p*-value (between male & female groups)	<0.05^*^	0.372^*^	<0.01^*^	

^a^Mean ± SD. ^b^Geometric mean (GSD) for log-transformed value

^*^ *p*- and ^†^ *p*-values were obtained by independent sample *t*-test and Chi-square test, respectively

The levels of DBP and SBP of the subjects in the arsenic-endemic areas were significantly (*p* < 0.001) higher than those of the subjects in the non-endemic area, as we reported [18, 35, 37]. Accordingly, the percentage of hypertensive subjects was also higher in the arsenic-endemic areas compared to the non-endemic area. When the levels of DBP and SBP and the percentage of hypertensive subjects were compared between males and females within the arsenic endemic areas, the SBP levels and the percentage of hypertensive patients were significantly higher in the females compared to the males. The differences in DBP and SBP between the arsenic-endemic and non-endemic areas were much greater in the females than the males.

As shown in Table 2, no significant differences in LINE-1 methylation levels were observed in the male subjects between those living in the arsenic-endemic areas and the non-endemic area. In the female subjects, however, the average levels of LINE-1 methylation were 64.23 ± 2.38 and 65.92 ± 1.66 in the arsenic-endemic and non-endemic areas respectively, and the difference was significant (*p* < 0.01). These data suggest that the association between arsenic exposure and LINE-1 hypomethylation is more pronounced in females than males.

Table 3 shows the results of the multiple linear regression analyses regarding the association between arsenic exposure and LINE-1 methylation levels. For all subjects, the non-adjusted associations between arsenic exposure and LINE-1 methylation were significantly negative (*R* ^2^ = 0.033,95% CI = −0.241, −0.043, *p* < 0.01 for water arsenic; *R* ^2^ = 0.019,95% CI = −0.378, −0.014, *p* < 0.05 for hair arsenic; and *R* ^2^ = 0.054,95% CI = −0.595,−0.179, *p* < 0.001for nail arsenic). After adjustment for age, BMI and smoking, the associations between arsenic exposure and LINE-1 methylation levels remained significant (*R* ^2^ = 0.057,95% CI = −0.238, −0.038, *p* < 0.01 for water arsenic; *R* ^2^ = 0.045,95% CI = −0.382, −0.014, *p <* 0.05 for hair arsenic; and *R* ^2^ = 0.079,95% CI = −0.591, −0.175, *p* < 0.001 for nail arsenic).

**Table 3:** Associations of arsenic exposure metrics with LINE-1 methylation levels

As-exposure	Dependent variable LINE-1 methylation (%)					
	All subjects^a^		Males^b^		Females^c^	
	Unadjusted	Adjusted	Unadjusted	Adjusted	Unadjusted	Adjusted
Water As						
R^2^	0.033	0.057	0.003	0.018	0.079	0.085
ΔR^2^	0.024		0.015		0.006	
95% CI	−0.241,−0.043	−0.238,−0.038	−0.161, 0.085	−0.178,0.076	−0.410,−0.088	−0.407,−0.081
*p*-value	<0.01	<0.01	0.539	0.428	<0.01	<0.01
Hair As						
R^2^	0.019	0.045	0.003	0.019	0.043	0.053
ΔR^2^	0.009		0.016		0.010	
95% CI	−0.378,−0.014	−0.382,−0.014	−0.282,0.150	−0.318,0.129	−0.662,−0.038	−0.664,−0.037
*p*-value	<0.05	<0.05	0.545	0.406	<0.05	<0.05
Nail As						
R^2^	0.054	0.079	0.016	0.031	0.100	0.106
ΔR^2^	0.025		0.015		0.006	
95% CI	−0.595,−0.179	−0.591,−0.175	−0.469,0.079	−0.490,0.066	−0.862,−0.236	−0.858,−0.224
*p*-value	<0.001	<0.001	0.162	0.134	<0.01	<0.01

Log-transformed values of arsenic concentrations were used

^a^Adjusted for age, sex, BMI and smoking status

^b^Adjusted for age, BMI and smoking status

^c^Adjusted for age and BMI

Since we observed a sex-based difference in LINE-1 methylation (Table 2), we performed sex-specific analyses. Intriguingly, we found that the drinking water, hair and nail arsenic concentrations of the female subjects showed significant inverse associations with LINE-1 methylation both before (*R* ^2^ = 0.079,95% CI = −0.410, −0.088, *p* < 0.01 for water arsenic;*R* ^2^ = 0.043,95% CI = −0.662,−0.038, *p* < 0.05 for hair arsenic; and *R* ^2^ = 0.100,95% CI = −0.862, −0.236, *p* < 0.01 for nail arsenic) and after (*R* ^2^ = 0.085,95% CI = −0.407, −0.081, *p* < 0.01 for water arsenic;*R* ^2^ = 0.053,95% CI = −0.664, −0.037, *p <* 0.05 for hair arsenic; and *R* ^2^ = 0.106,95% CI = −0.858, −0.224, *p* < 0.01 for nail arsenic) adjustment for covariates. No significant associations were observed in the male subjects.

Table 4 shows the associations of arsenic exposure with BP with and without adjustment for LINE-1 methylation levels through multivariate linear regression analyses. In all subjects and in the female subjects, the water, hair and nail arsenic concentrations showed significant associations with DBP and SBP without adjustment for LINE-1 methylation. However, after adjustment with LINE-1 methylation, the associations between arsenic exposure and DBP and SBP were weakened in all subjects, and the associations completely disappeared in the female subjects.

**Table 4:** Associations of arsenic exposure and LINE-1 methylation levels with BP through multivariate regression analyses

Independent variables	Dependent variable DBP											
	All subjects				Males				Females			
	Model 1		Model 2		Model 1		Model 2		Model 1		Model 2	
	β (95% CI)	*p*-value	β (95% CI)	*p*-value	β (95% CI)	*p*-value	β (95% CI)	*p*-value	β (95% CI)	*p*-value	β (95% CI)	*p*-value
Water As	0.742 (0.189,1.294)	<0.01	0.608 (0.053,1.163)	<0.05	0.513 (−0.159,1.184)	0.133	0.509 (−0.166,1.184)	0.138	1.009 (0.078,1.941)	<0.05	0.614 (−0.324,1.551)	0.197
LINE-1	−1.084 (−1.787,−0.382)	<0.01	−0.944 (−1.652,−0.235)	<0.01	−0.138 (−1.123,0.846)	0.781	−0.097 (−1.079,0.884)	0.844	−1.786 (−2.808,−0.765)	<0.01	−1.591 (−2.652,−0.529)	<0.01
Hair As	1.324 (0.315,2.333)	<0.05	1.133 (0.129, 2.138)	<0.05	0.926 (−0.255,2.107)	0.123	0.919 (−0.268,2.107)	0.128	1.870 (0.097,3.643)	<0.05	1.301 (−0.444,3.046)	0.142
LINE-1			−0.976 (−1.679,−0.272)	<0.01			−0.097 (−1.078,0.884)	0.845			−1.625 (−2.664,−0.586)	<0.01
Nail As	1.972 (0.810,3.134)	<0.01	1.642 (0.458, 2.825)	<0.01	1.847 (0.357,3.338)	<0.05	1.850 (0.341,3.358)	<0.05	2.068 (0.240,3.897)	<0.05	1.209 (−0.656,3.074)	0.202
LINE-1			−0.854 (−1.567,−0.141)	<0.05			0.012 (−0.962,0.985)	0.981			−1.567 (−2.640,0.493)	<0.01
	Dependent variable SBP											
Water As	1.098 (0.257,1.939)	<0.05	0.888 (0.044,1.732)	<0.05	0.481 (−0.472,1.433)	0.320	0.470 (−0.487,1.428)	0.333	1.678 (0.213,3.143)	<0.05	1.140 (−0.350,2.630)	0.132
LINE-1	−1.687 (−2.755,−0.619)	<0.01	−1.481 (−2.559,− 0.403)	<0.01	−0.308 (−1.696,1.080)	0.661	−0.270 (−1.661,1.120)	0.701	−2.527 (−4.155,−0.899)	<0.01	−2.163 (−3.850,−0.476)	<0.05
Hair As	1.904 (0.367,3.441)	<0.05	1.603 (0.074, 3.133)	<0.05	1.257 (−0.410,2.925)	0.138	1.241 (−0.435,2.916)	0.145	2.562 (−0.248,5.371)	0.074	1.753 (−1.037,4.543)	0.216
LINE-1			−1.533 (−2.603,−0.426)	<0.01			−0.252 (−1.636,1.132)	0.719			−2.309 (−3.970,−0.649)	<0.01
Nail As	2.930 (1.161,4.700)	<0.01	2.408 (0.608, 4.209)	<0.01	2.368 (0.257,4.480)	<0.05	2.345 (0.209,4.482)	<0.05	3.294 (0.412,6.176)	<0.05	2.119 (−0.849, 5.086)	0.160
LINE-1			−1.349 (−2.433, 0.265)	<0.05			−0.118 (−1.496,1.261)	0.866			−2.142 (−3.850,−0.434)	<0.05

Log-transformed values of arsenic concentrations were used. Before adjustment (Model 1) and after adjustment (Model 2) with LINE-1 methylation levels

Additional file 1 shows the associations between the arsenic exposure metrics and LINE-1 methylation levels with BP after adjustment for age, sex, BMI and smoking. The adjusted association between arsenic exposure levels and BP, and that between LINE-1 methylation levels and BP were found to be significant.

## Discussion

In this study, we explored the association between arsenic exposure, as measured by drinking water, hair and nail arsenic concentrations, and LINE-1 methylation levels through a cross sectional study recruiting human subjects from arsenic-endemic and non-endemic areas in Bangladesh. We also examined whether the altered methylation levels were associated to arsenic exposure-related elevated levels of BP. The results demonstrated that the LINE-1 methylation levels were significantly lower among individuals living in arsenic-endemic areas compared to those in non-endemic areas (Table 2). The drinking water, hair and nail arsenic concentrations of the female subjects showed significant negative associations with LINE-1 methylation levels (Table 3). On the contrary, no significant associations of arsenic exposure metrics with LINE-1 methylation levels were observed in the males, suggesting that the apparent associations between arsenic exposure and LINE-1 hypomethylation in all subjects may be a reflection of the significant association in females (Table 3). In addition, the arsenic exposure metrics in the females showed significant positive associations with BP. Intriguingly, interaction analyses revealed that the arsenic-related elevated levels of BP were correlated with LINE-1 hypomethylation levels (Table 4).

A good number of studies using cell lines and experimental animals have been conducted to evaluate the effects of arsenic exposure on global DNA methylation [39–48]. Human studies have also revealed the effects of arsenic exposure on DNA methylation status, but the specific alterations are not consistently observed. By conducting a methylation incorporation assay, Pilsner et al. [24] showed that the plasma arsenic and urinary arsenic levels of their subjects were negatively correlated with the extent of [^3^H]-methyl incorporation, suggesting that arsenic exposure was positively associated genomic methylation levels of blood leukocyte DNA. In another study, the same group (2009) [25] reported that arsenic-exposed individuals with skin lesions had lower levels of methylation in DNA than the arsenic-exposed individuals without skin lesions. Majumdar et al. [21] observed an association between genome-wide hypermethylation and drinking water arsenic levels, in a population of only 64 individuals. In a gene-specific analysis, Hossain et al. [30] found that urinary arsenic was positively associated with the methylation of a tumor suppressor gene, *p16*. In the same study, they did not observed an association between urinary arsenic and LINE-1 methylation. In a study of neonates in Bangladesh, Kile et al. [31] identified a positive association between maternal urinary total arsenic and LINE-1 methylation levels in both the mother and newborns. Pilsner et al. [29] obtained similar results in their study of mother-newborn pairs in Bangladesh; however, in their sex-specific analyses, the associations between arsenic exposures and ALU, LINE-1 and LUMA were positive among the male newborns but negative among the female newborns (although the results were not significant). In contrast, Broberg et al. [32] reported that early prenatal arsenic exposure appears to decrease DNA methylation in boys but not in girls. It is not yet clear how long the alteration in the methylation status in DNA is stable or what the consequences of the neonatal alteration of DNA methylation will be in later life. Niedzwiecki et al. [23] reported that arsenic exposure was not associated with global 5-methyl cytosine (MC) levels or 5-hydroxyl methyl cytosine (hmC) levels in their complete subject population, but in their sex-specific analyses, they observed a positive association between arsenic exposure and global 5-hmC levels in the males and a negative association in the females. The female-specific LINE-1 hypomethylation revealed in our present study partially agrees with the findings of Niedzwiecki et al.’s study. The discrepancies in the results from one study to another may be due to differences in arsenic doses, exposure times, the repetitive elements of DNA targeted for measuring global methylation levels, and nutritional or other confounding factors influencing the effects of arsenic exposure. Most of the previous studies did not demonstrate the biological consequences of the relationships between arsenic exposure and changes in DNA methylation, and thus there are great uncertainties regarding the pathophysiological consequences of the arsenic-induced alteration of methylation status in DNA.

Against this background, our present data demonstrating an association between arsenic exposure and reduced levels of LINE-1 methylation and their interactions with elevated levels of BP are noteworthy. Recent studies have shown a link between the decreased methylation levels in global and specific DNAs with CVD-related events [5, 49]. Castro et al. [50] reported lower DNA methylation levels in peripheral blood leukocytes from patients with atherosclerotic disease. It was also reported that a lower blood LINE-1 methylation level is a predictive factor for the incidences and mortalities of ischemic heart disease and stroke [5]. Bellavia et al. [51] showed an association between particulate-matter-mediated hypomethylation and BP. Similar to the results of our previous research [18, 35, 37], in the present study we also observed that the average levels of BP and the percentage of hypertensive patients were higher in the arsenic-endemic areas compared to the non-endemic area, and the average levels of SBP and the percentage of hypertensive patients were higher in the females than the males in the arsenic-endemic areas (Table 2).

Intriguingly, in our multivariate regression analysis, the significance of the associations of arsenic exposure metrics with DBP and SBP disappeared in the female subjects after the adjustment for LINE-1 methylation levels, indicating that LINE-1 hypomethylation might be involved in an arsenic-related elevation of BP (Table 4). These results are in line with those of studies that showed a link between LINE-1 hypomethylation and CVD, especially hypertension [16, 50, 51]. It should be noted here that sex differences are also known to be present in other diseases caused by chronic exposure to arsenic. For example, women have been found to have higher risks of arsenic-related kidney, lung and bladder cancers as well as diabetes compared to men [14, 52–54]. The higher susceptibility of arsenic-exposed females to hypomethylation in DNA observed in the present study may also be associated with the higher incidences of those diseases in females compared to males.

Demographic and lifestyle characteristics such as age, sex, BMI, smoking, and alcohol consumption may be associated with global DNA methylation. However, the reports- regarding the effects of these characteristics are inconsistent [17, 26, 55]. In our regression analyses, we did not consider alcohol consumption as a variable because none of the subjects admitted to drinking alcohol. Before and after the adjustment for covariates (age, sex, BMI and smoking), significance levels of the associations between arsenic exposure and LINE-1 hypomethylation were not considerably different in the females, which indicated that age, sex and BMI had little or almost no effects on the associations. These results are consistent with those reported by Cash et al. [56]. In our study, the majority (65.60%) of the male subjects were not smokers and no females were smokers. In the selection process of the present study, we excluded older individuals (>60 years old). The narrow ranges of age (IQR: 28–43.70 years) and BMI (IQR: 18.25–22.57) and the smaller number of smokers may explain why we did not observe any confounding effects of those variables on LINE-1 methylation status.

Since a variety of factors are involved in the development of hypertension, it seems unlikely that the epigenetic regulation of global genes is a sole factor causing hypertension in individuals living in arsenic-endemic areas. To eliminate the confounding effects of age, sex, BMI, and smoking on the associations of arsenic exposure and LINE-1 methylation with BP, we performed further regression analyses (Additional file 1), and we found that arsenic exposure and LINE-1 hypomethylation retained their significant association with BP after the adjustment for those variables. These results suggest that arsenic exposure and LINE-1 hypomethylation may be important contributors to the elevation of BP.

It remains unknown why females are more susceptible to arsenic-induced LINE-1 hypomethylation than males. One possibility is that higher levels and more consistent exposure to arsenic through drinking water in females than males may make females more susceptible to LINE-1 hypomethylation. Our female subjects (most of whom are housewives) may depend more on arsenic-contaminated tube-well water at home than the males (most of whom are farmers), who work outside their homes. The average concentrations of drinking water in arsenic-endemic areas were higher in the females than males, although the significance of the difference was borderline (*p* = 0.056) (Table 2). However, this result may not be enough to draw a conclusion, because the differences in hair and nail arsenic concentrations — which are more important indicators of the body’s accumulation of arsenic — were not significant between the males and females. Another possible explanation for female susceptibility to LINE-1 hypomethylation is the effects of sex hormones. Cell line experiments demonstrated that the methylation levels of LINE-1 were decreased by treatment with estradiol but not with dihydrotestosterone [57]. Arsenic exposure has been reported to have endocrine-disruptive effects [58, 59], and it may thus be likely that arsenic causes hypomethylation through its endocrine-disrupting activity.

The strength of the present study was that we examined three types of exposure metrics (drinking water, hair and nail arsenic concentrations), and all indicators including external (drinking water) and internal (hair and nail) exposure metrics showed significant negative associations with the levels of LINE-1 methylation in the female subjects (Tables 3). Hypomethylation in the LINE-1 sequence across the three exposure metrics may exclude the potential bias in the observed associations.

However, our study has some limitations that should be carefully evaluated. First, although we detected significant differences in hypomethylation in LINE-1 between the residents of the arsenic-endemic and non-endemic areas, the absolute values were very small. The differences in LINE-1 methylation levels in the females were within 2% between the arsenic-endemic and non-endemic areas (Tables 2). Similar to our results, however, small but significant differences in global DNA methylation were reported to be associated with the risk of head and neck squamous cell carcinoma (74.7% in cases vs. 75.3% in controls [60] and Alzheimer disease (AD);AD: 83.6%5mC, volunteers: 83.1% 5mC, *p* = 0.05) [61]. Moreover, recently published data showed that a small reduction in LINE-1 methylation significantly increased the risk of coronary heart disease in a Chinese population [17]. All of these results support the notion that a small difference in global genomic DNA methylation could be involved in the pathogenesis of several diseases including CVD.

Second, the nutritional status of our subjects which were not considered in this study may have affected the levels of global DNA methylation. It was suggested that the folate nutritional status may alter the methylation of DNA [24]. Third, we examined the LINE-1 methylation in blood leukocyte DNA, but it remains unclear whether the leukocytes’ DNA reflects the effects of arsenic exposure on other organs such as skin, liver, bladder, kidney, spleen and lung. Although growing evidence has suggested global DNA hypomethylation in peripheral blood leukocytes as a biomarker for cancer and CVD, an organ-specific DNA methylation status may be more important to understand organ-specific effects and health outcomes caused by chronic exposure to arsenic.

Because of their wide distribution in genomic DNA, the methylation levels of LINE-1 have been used as a proxy for global DNA methylation status [62–64]. Methylation at LINE-1 repeats is gaining increasing attention as a surrogate marker to distinguish normal from pathological disease tissue [57]. Decreased methylation of LINE-1 sequences in blood leukocyte DNA has been documented in many human cancers and other chronic diseases [12–16] Therefore, LINE-1 hypomethylation in blood leukocyte-derived DNA caused by chronic exposure to arsenic may be one of the potential epigenetic mechanisms of arsenic-induced chronic diseases, including hypertension.

## Conclusions

Our data demonstrated that chronic exposure to arsenic is significantly associated with global DNA hypomethylation measured in LINE-1 repeats in the blood leukocyte DNA; this association is more pronounced in females than males. We also observed a significant interaction of arsenic-related elevated levels of BP with LINE-1 hypomethylation, which indicates the possible involvement of LINE-1 hypomethylation in chronic arsenic exposure-related hypertension in females. A large prospective study is required to test these observations.

## Additional file

Additional file 1: Adjusted associations of arsenic exposure metrics and LINE-1 methylation levels with BP through multivariate regression analyses. (DOC 67 kb)

## Abbreviations

BP: Blood pressure
CVD: Cardiovascular disease
DBP: Diastolic blood pressure
LINE-1: Long interspersed nuclear element-1
SBP: Systolic blood pressure
