# Supplementary material for: Chronic exposure to arsenic, LINE-1 hypomethylation, and blood pressure: a cross-sectional study in Bangladesh
Source: Environ Health. 2017 Mar 7;16:20. doi: 10.1186/s12940-017-0231-7 (PMC5341433; doi:10.1186/s12940-017-0231-7)
Supplement: Additional file 1: — Adjusted associations of arsenic exposure metrics and LINE-1 methylation levels with BP through multivariate regression analyses. (DOC 67 kb) [file 12940_2017_231_MOESM1_ESM.doc]

Additional file 1 Adjusted associations of arsenic exposure metrics and LINE-1 methylation levels with BP through multivariate regression analyses

| Independent variables | **Dependent variable** | | | |
| --- | --- | --- | --- | --- |
|  | **DBP** | | **SBP** | |
|  | **All Subjects^a^** | | | |
|  | β(95% CI) | *p*-value | β(95% CI) | *p*-value |
| Water As | 0.837 (0.305,1.368) | <0.01 | 1.207 (0.417,1.997) | <0.01 |
| Hair As | 1.509 (0.537,2.482) | <0.01 | 2.188 (0.743,3.633) | <0.01 |
| Nail As | 2.061 (0.952,3.170) | <0.001 | 3.039 (1.392,4.687) | <0.001 |
| LINE-1 | −0.987 (−1.669,−0.305) | <0.01 | −1.381 (−2.395,−0.366) | <0.01 |
|  | **Males^b^** | | | |
| Water As | 0.734(0.063,1.405) | <0.05 | 0.908 (−0.022,1.837) | 0.056 |
| Hair As | 1.301 (0.116,2.486 | <0.05 | 1.994 (0.365,3.622) | <0.05 |
| Nail As | 2.096 (0.633,3.559) | <0.01 | 2.877 (0.857,4.897) | <0.01 |
| LINE-1 | −0.123 (−1.097,0.850) | 0.802 | −0.192 (−1.536,1.151) | 0.777 |
|  | **Females**^c^ | | | |
| Water As | 0.847(−0.012,1.707) | 0.053 | 1.456(0.111,2.818) | <0.05 |
| Hair As | 1.907 (0.296,3.517) | <0.05 | 2.648(0.088,5.208) | <0.05 |
| Nail As | 1.819 (0.138,3.499) | <0.05 | 2.970 (0.317,5.622) | <0.05 |
| LINE-1 | −1.565 (−2.505,−0.624) | <0.01 | −2.189 (−3.691,−0.686) | <0.01 |

Log-transformed values of As exposure metrics were used. ^a^Adjusted for age, sex, BMI and smoking status. ^b^Adjusted for age, BMI and smoking status. ^c^Adjusted for age and BMI.
